# Potential Interaction of Green Tea Extract with Hydrochlorothiazide on Diuretic Activity in Rats

**DOI:** 10.1155/2014/273908

**Published:** 2014-11-05

**Authors:** Manodeep Chakraborty, Jagadish V. Kamath, Ananya Bhattacharjee

**Affiliations:** ^1^Bhagwant University, Ajmer 305004, India; ^2^Department of Pharmacology, Shree Devi College of Pharmacy, Mangalore 574142, India

## Abstract

Treatment of ischemic hypertensive patients with hydrochlorothiazide can precipitate cardiac arrhythmias. The present study was undertaken to evaluate the diuretic potential of green tea alone and its effects on hydrochlorothiazide in interactive groups. Rats were treated with high (500 mg/kg, p.o.) and low (100 mg/kg, p.o.) dose of green tea extract in alone and interactive groups for 30 days. Standard, high, and low dose interactive groups received hydrochlorothiazide (10 mg/kg, p.o.) on the day of experiment. Effect of different treatments was that assessed by evaluating diuretic action, diuretic activity, percentage of saline load excreted, and sodium and potassium levels in urine. Green tea in both high and low doses showed significant diuretic potential and when it is combined with hydrochlorothiazide resulted in significant improvement in the activity compared to hydrochlorothiazide alone treated group. It can be concluded that green tea extract when combined with hydrochlorothiazide showed significant increase in diuretic activity. Most important observation of the present study is even though the combination increases the diuretic potential, it is responsible for decrease in urinary potassium loss.

## 1. Introduction

Diuretic compounds that stimulate the excretion of water are potentially useful in most of the disorders including those exhibiting oedema, such as congestive heart failure, nephritis, toxemia of pregnancy, premenstrual tension, and hypertension. The presently available diuretics such as thiazides and loop diuretics exhibit various adverse effects, such as electrolyte imbalance and metabolic alterations. Some of the diuretics are derived from medicinal plants and a vast number of medicinal plants mentioned in ayurvedic system of medicine are known to possess diuretic properties such as* Abelmoschus esculentus*,* Bacopa monnieri*,* Barbara vulgaris*, and* Cissampelos pareira* [1].

Diuretics, in particular hydrochlorothiazide (HCTZ), are in close watch in the management of hypertension in patients with ischemic heart diseases. Thiazides are responsible for the occurrence of hypokalemia by increase in urinary potassium loss [2]. It has been documented that even mild or moderate hypokalemia increases the risks of morbidity and mortality in patients with cardiovascular disease [3]. Several studies have now demonstrated that a significant incidence of ventricular ectopic activity with diuretic-induced hypokalemia is responsible for sudden death [4]. Apart from plasma potassium levels associated with thiazides, the activation of the renin-angiotensin-aldosterone system and, in particular, the stimulation of mineralocorticoid receptors are key determinants in the inflammatory process and the progression of cardiovascular disease [5].

Green tea is one of the most consumed beverages in the world. It is obtained from the nonfermented leaves of* Camellia sinensis* belonging to family Theaceae, which contains more catechins than black tea or oolong tea. Due to the presence of high levels of catechin, certain minerals and vitamins increase the antioxidant potential of this type of tea. Since ancient times, green tea has been considered as a healthful beverage by the traditional Chinese medicine. Recent studies have reported that green tea may contribute to the reduction in risk of cardiovascular diseases and some forms of cancer, as well as to the promotion of oral health. It has antibacterial, antiviral, neuroprotective, and antifibrotic properties. It is reported that green tea gives protection from solar ultraviolet rays and increases bone mineral density [6, 7].

Till now no study has been conducted to demonstrate the diuretic effect of green tea. Hot water infusion of Sri Lankan black tea was reported for having potential diuretic activity and presence of caffeine has been stated as main key component for the activity [8].

So the present study has been designed to evaluate the effect of green tea alone and in combination with hydrochlorothiazide on diuretic activity.

## 2. Materials and Methods

### 2.1. Chemicals

All chemicals used were of analytical grade and purchased from standard companies. Pure sample of hydrochlorothiazide was gifted by Bangalore Test House (Bangalore, India). Biochemical kits were procured from Crest Biosystems (Goa, India).

### 2.2. Experimental Animals

Healthy adult Wistar albino rats of either sex weighing 175–250 g, aged four weeks, were procured from Animal House, Shree Devi College of Pharmacy, Mangalore. Rats were housed in polypropylene cages and maintained under standardized condition (12 h L: D cycles, 25° ± 5°C) with paddy husk bedding at the Central Animal House, Shree Devi College of Pharmacy, Mangalore. Animals were provided with standard pellet food and had free access to purified drinking water. The guidelines of Committee for the Purpose of Control and Supervision of Experiments on Animals (CPCSEA), Ministry of Social Justice and Empowerment, Government of India, were followed and prior permission was sought from the Institutional Animal Ethics Committee for conducting the study (SDCP/IAEC-19/2012-13).

### 2.3. Plant Materials

Green tea (*Camellia sinensis*) leaves were purchased in the month of June, 2013, from the local market of Mangalore bearing the brand name GREEN TEA (manufactured by New hilltop traders, Vandiperiyar, Kerala). The authentication was done by Dr. Neoline J. Pinto, H.O.D., Department of Botany, St. Agnes College, Mangalore (SAC/MNG/SMP/Drug/2013-06/52). The leaves were milled with hammer mill and passed through 1 mm mesh screen. One hundred grams of leaves were boiled with 1 liter of distilled water for 10 min at 70°C. The heated solution was filtered, evaporated under vacuum, and freeze dried to get a thick gummy mass [9]. The yield was found to be 24.76% (W/W).

### 2.4. Phytochemical Estimations of the Extract

The aqueous extract of green tea was subjected to qualitative analysis for the following organic plant constituents: alkaloids, proteins amino acids, anthraquinones, flavonoids, carbohydrates, saponins, tannins, steroids, triterpenoids, and cardiac glycosides [10, 11] (Table 1).

### 2.5. Acute Toxicity Study

Acute toxicity study was carried out according to OPPTS (Office of Prevention, Pesticide and Toxic Substance) guidelines following the limit test procedure [12].

Mice were fasted overnight prior to the studies and then divided into two groups of three each. Test dose of 2 g/kg body weight and 5 g/kg body weight was given orally to either group of mice and then observed for 72 hours for mortality. 1/10th and 1/50th of the maximum safe dose corresponding to 500 and 100 mg/kg orally were selected as high and low doses, respectively.

### 2.6. Experimental Protocol

Rats were divided into the following six groups consisting of eight animals each:

 Group I: vehicle (1 mL/kg, p.o. for 30 days),
 Group II: hydrochlorothiazide (HCTZ) 10 mg/kg on the day of experiment, p.o.,
 Group III: green tea extract (GTE) 100 mg/kg for 30 days, p.o.,
 Group IV: green tea extract (GTE) 500 mg/kg for 30 days, p.o.,
 Group V: green tea extract (GTE) 100 mg/kg for 30 days + HCTZ,
 Group VI: green tea extract (GTE) 500 mg/kg for 30 days + HCTZ.

Prophylactically treated animals were fasted overnight with water allowed* ad libitum*. The next day morning rats were given orally 25 mL/kg of normal saline solution, and, immediately after normal saline administration, the rats were placed individually in a modified funnel having a wire mesh and fitted with a graduated test tube. In HCTZ incorporated groups, HCTZ was given orally as a fine homogenized suspension in a volume of 25 mL/kg of normal saline solution. Urine excreted for the next 5 h was collected and the total volume of urine for each rat was compared with the volume of urine produced after the administration of normal saline. The volume of urine excreted during 5 h for each animal in the group is expressed as the percent of the liquid (normal saline) administered. This percentage gives a measure of urinary excretion independent of the animal weight. The ratio of urinary excretion in the test group to urinary excretion in the control group is used as a measure of the diuretic action for the given dose of the drug. As the diuretic action is prone to variability, a parameter known as diuretic activity was calculated instead. To obtain the diuretic activity, the diuretic action of the test groups (green tea extract) was compared with that of the standard (HCTZ).

Percentage of saline load excreted = volume of urine/volume of saline load × 100.

Urinary excretion = total urinary output/total liquid administered × 100.

Diuretic action = urinary excretion of treated group/urinary excretion of control group.

Diuretic activity = diuretic action of test drug/diuretic action of standard drug.

Urinary Na^+^ and K^+^ contents were analyzed by auto analyzer [2].

### 2.7. Statistical Analysis

Results are expressed as mean +/− SEM. Statistical significance was assessed using one-way analysis of variance (ANOVA) followed by Tukey-Kramer multiple comparison tests. *P* < 0.05 was considered significant.

## 3. Results

### 3.1. Preliminary Phytochemical Investigation

The preliminary phytochemical investigation of aqueous extract of the green tea showed the presence of alkaloids, flavonoids, steroids, tannins, proteins, amino acids, carbohydrates, reducing sugar, and deoxysugars and indicated the absence of terpenoids, saponins, glycosides, and anthraquinones. The percentage yield of GTE was found to be 24.76%.

### 3.2. Effect on Electrolyte Excretion in Urine

#### 3.2.1. Effect on Sodium Ion Concentration in Urine

Significant increase in Na^+^ ion excretion in urine compared with normal control was obtained in all treatments, with HCTZ inducing the maximum excretion either alone (120.82 ± 4.45, *P* < 0.001) or in association with GTE (138.92 ± 3.83 and 159.39 ± 4.39, *P* < 0.01, for GTE100 + HCTZ and GTE-500 + HCTZ, resp.).

**Figure 1:**
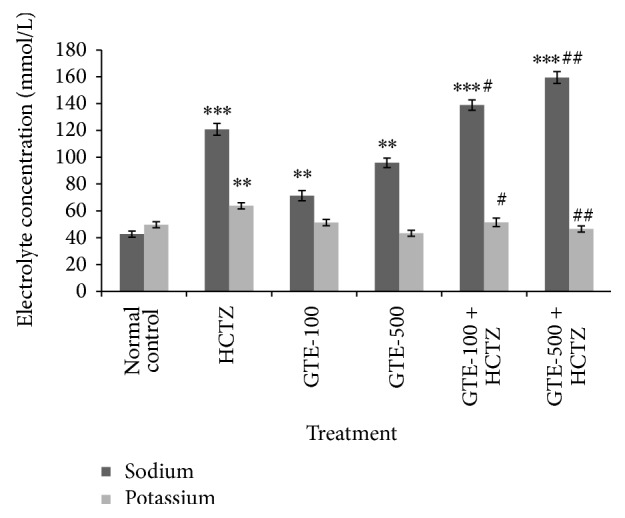
Effect on electrolyte excretion in urine. All values are mean ± SEM, *n* = 8, ^***^ *P* < 0.001, ^**^ *P* < 0.01 when compared to normal control; ^##^ *P* < 0.01, ^#^ *P* < 0.05 compared to hydrochlorothiazide. GTE-100 (green tea extract, 100 mg/kg), GTE-500 (green tea extract, 500 mg/kg), and HCTZ (hydrochlorothiazide, 10 mg/kg).

GTE-100 + HCTZ showed significant (*P* < 0.05) increase but in case of GTE-500 + HCTZ there was significant (*P* < 0.01) increase in Na^+^ ion concentration compared to HCTZ alone treated group (Table 2) (Figure 1).

#### 3.2.2. Effect on Potassium Ion Concentration in Urine

HCTZ treated group showed significant (*P* < 0.01) increase in K^+^ concentration in urine compared to normal control.

GTE-100 + HCTZ showed significant (*P* < 0.05) whereas in case of GTE-500 + HCTZ significant (*P* < 0.01) decrease in K^+^ concentration in urine has been observed compared to HCTZ group (Table 2) (Figure 1).

#### 3.2.3. Effect on Percentage Saline Load Excretion and Diuretic Action

All treatments showed significant increases in the percentage of saline load excretion compared to normal control.

GTE-500 + HCTZ treated group is responsible for significant (*P* < 0.05) increase in percentage saline load excretion and diuretic action compared to HCTZ alone treated group (Table 3).

#### 3.2.4. Effect on Diuretic Activity

Diuretic activity for GTE-100 and GTE-500 was 0.60 and 0.75, respectively, whereas for GTE-100 + HCTZ and GTE-500 + HCTZ it was found to be 1.08 and 1.28, respectively (Table 2).

## 4. Discussion

The aim of the present study was to investigate the effect of green tea alone and with combination of hydrochlorothiazide (HCTZ) on diuretic activity. Observed results suggested that GTE (100 and 500 mg/kg, p.o.) in both doses showed significant diuretic activity. Further results suggested that green tea when combined with HCTZ showed significant increase in diuretic potential but urinary loss of K^+^ level has been decreased significantly compared to HCTZ alone treated group.

Diuretic action of HCTZ is associated with inhibition of Na^+^/Cl^−^ symporter (cotransporter) system in the distal convoluted tubule, by competing for the Cl^−^ binding sites, and increase in the excretion of Na^+^ by inhibiting Na^+^ reabsorption, thereby increasing in urinary output. Indirectly, the diuretic action of HCTZ reduces plasma volume, with a consequent increase in urinary potassium loss, plasma renin activity, and aldosterone secretion and decrease in serum potassium [13, 14].

In this present study HCTZ demonstrated significant increase in urinary Na^+^ and K^+^ levels. HCTZ also demonstrated significant increase in diuretic action compared to normal control.

GTE (100 and 500 mg/kg, p.o.), in both high and low doses, also demonstrated significant increase in urinary Na^+^ and diuretic activity compared to control with no significant urinary K^+^ loss.

The main flavanols and purine alkaloids present in green tea are responsible for inhibition of angiotensin-converting enzyme (ACE) activity. Green tea inhibits ACE activity by mixed inhibitor mechanism [15, 16]. ACE inhibition may be one of the prime reasons to demonstrate diuretic activity and increase in urinary Na^+^ level [17]. Apart from that green tea is also responsible for inhibition of carbonic anhydrase activity. Green tea causes increase in glomerular filtration rate by increase in renal blood flow and cardiac output which may be a contributor to diuretic activity [8]. Findings of the present study establish these facts by significant increase in diuretic activity of GTE in a dose dependent manner. GTE when combined with HCTZ is able to show incremental diuretic potency compared to HCTZ alone treated group.

ACE inhibition associated with GTE causes decrease in aldosterone activity and increase in potassium retention [17]. In accordance with that results showed no significant potassium loss with GTE treatment. In combination groups also GTE significantly reduced potassium loss compared to HCTZ alone treated group. The anti-inflammatory property of GTE also can be a contributing factor to demonstrating diuretic activity [18].

The groups treated with low and high dose of GTE along with HCTZ showed a significant increase in diuretic potential by increasing Na^+^ ion excretion, percentage of saline load excreted, diuretic action, and diuretic activity parameters. Interestingly K^+^ loss in combination groups has been decreased significantly compared to HCTZ alone treated group. This observation is very important as green tea is able to reduce the HCTZ induced hypokalemia. Moreover, in the combination groups GTE causes increase in the efficacy of HCTZ in a dose dependent manner.

## 5. Conclusion

From this present study it can be concluded that GTE when combined with HCTZ showed significant increase in diuretic activity. Most important observation of the present study is that even though the combination increases the diuretic potential, it is responsible for decrease in urinary potassium loss which decreases the chances of hypokalemia. This study can be proved beneficial for the ischemic hypertensive patients where HCTZ dose can be reduced and is possible to minimize the associated side effects in presence of GTE.

## Figures and Tables

**Table 1:** Qualitative analysis of the extract.

Different plant constituents	Tests
Test for alkaloids	Mayer's test, Dragendroff's test, Wagner's test, and Hager's test
Test for proteins and amino acids	Millon's test, Biuret test, and ninhydrin test
Test for anthraquinones	Modified Borntrager's test
Test for flavonoids	Ferric chloride test and lead acetate test
Test for carbohydrates	Molisch's test
Test for reducing sugars	Fehling's test and Benedict's test
Test for saponins	Foam formation test
Test for tannins	Ferric chloride test and lead acetate test
Test for steroids, triterpenoids, and cardiac glycosides	Liebermann-Burchard test, Salkowski test, Noller's test, Legal's test, Baljet test, and Keller Kiliani's test

**Table 2:** Effect on electrolyte excretion in urine.

Treatment	Electrolyte concentration (mmol/L)	
	Na^+^	K^+^
Normal control	42.72 ± 2.29	49.65 ± 2.19
HCTZ	120.82 ± 4.45^***^	63.83 ± 2.29^**^
GTE-100	71.39 ± 3.81^**^	51.29 ± 2.34
GTE-500	95.83 ± 3.56^**^	43.39 ± 2.21
GTE-100 + HCTZ	138.92 ± 3.83^∗∗∗#^	51.48 ± 3.193^#^
GTE-500 + HCTZ	159.39 ± 4.39^∗∗∗##^	46.52 ± 2.32^##^

All values are mean ± SEM, *n* = 8, ^***^ *P* < 0.001, ^**^ *P* < 0.01 when compared to normal control; ^##^ *P* < 0.01, ^#^ *P* < 0.05 compared to hydrochlorothiazide. GTE-100 (green tea extract, 100 mg/kg), GTE-500 (green tea extract, 500 mg/kg), and HCTZ (hydrochlorothiazide, 10 mg/kg).

**Table 3:** Effect on percentage saline load excretion and diuretic action.

Treatment	Percentage of saline load excreted	Diuretic action	Diuretic activity
Normal control	61.65 ± 2.92	1.34	—
HCTZ	136.91 ± 4.39^***^	3.10^***^	—
GTE-100	86.28 ± 3.71^*^	1.89^*^	0.60
GTE-500	95.34 ± 3.27^*^	2.35^*^	0.75
GTE-100 + HCTZ	147.19 ± 5.11^***^	3.36^***^	1.08
GTE-500 + HCTZ	161.64 ± 5.16^∗∗∗#^	3.97^∗∗∗#^	1.28

All values are mean ± SEM, *n* = 8, ^***^ *P* < 0.001, ^*^ *P* < 0.05 when compared to normal control; ^#^ *P* < 0.05 compared to HCTZ. GTE-100 (green tea extract, 100 mg/kg), GTE-500 (green tea extract, 500 mg/kg), and HCTZ (hydrochlorothiazide, 10 mg/kg).

## References

[1] Sayana S. B., Khanwelkar C. C., Nimmagadda V. R. (2014). Evaluation of diuretic activity of alcoholic extract of roots of Cissampelos pareira in albino rats. *Journal of Clinical and Diagnostic Research*.

[2] Asdaq S. M., Inamdar M. N. (2009). The potential for interaction of hydrochlorothiazide with garlic in rats. *Chemico-Biological Interactions*.

[3] Wojtaszek E., Matuszkiewicz-Rowińska J. (2013). Hypokalemia. *Wiadomości Lekarskie*.

[4] Holland O. B. (1986). Potassium loss, ventricular irritability, and the risk of sudden death in hypertensive patients. *Drugs*.

[5] Armanini D., Bordin L., Clari G. (2012). Serum potassium, thiazides, aldosterone, and mineralocorticoid receptors. *Hypertension*.

[6] Cabrera C., Artacho R., Giménez R. (2006). Beneficial effects of green tea—a review. *Journal of the American College of Nutrition*.

[7] Brown M. D. (1999). Green tea (*Camellia sinensis*) extract and its possible role in the prevention of cancer. *Alternative Medicine Review*.

[8] Abeywickrama K. R. W., Ratnasooriya W. D., Amarakoon A. M. T. (2010). Oral diuretic activity of hot water infusion of Sri Lankan black tea (Camellia sinensis L.) in rats. *Pharmacognosy Magazine*.

[9] Bajerska J., Wozniewicz M., Jeszka J., Drzymala-Czyz S., Walkowiak J. (2011). Green tea aqueous extract reduces visceral fat and decreases protein availability in rats fed with a high-fat diet. *Nutrition Research*.

[10] Finar I. L. (1993). *Organic Chemistry*.

[11] Mukherjee P. K. (2002). *Quality Control of Herbal Drugs—An Approach to Evaluation of Botanicals*.

[12] Chakraborty M., Asdaq S. M. B. (2011). Interaction of Semecarpus anacardium L. with propranolol against isoproterenol induced myocardial damage in rats. *Indian Journal of Experimental Biology*.

[13] Jackson E. K., Hardmen J. C., Gilman A. G., Limbird L. E. (1996). Drugs affecting renal and cardiovascular function. *Goodman and Gilman’s the Pharmacological Basis of Therapeutics*.

[14] Field M. J., Stanton B. A., Giebisch G. H. (1984). Differential acute effects of aldosterone, dexamethasone, and hyperkalemia on distal tubular potassium secretion in the rat kidney. *Journal of Clinical Investigation*.

[15] Persson I. A.-L., Josefsson M., Persson K., Andersson R. G. G. (2006). Tea flavanols inhibit angiotensin-converting enzyme activity and increase nitric oxide production in human endothelial cells. *Journal of Pharmacy and Pharmacology*.

[16] Persson I. A.-L. (2012). The pharmacological mechanism of angiotensin-converting enzyme inhibition by green tea, Rooibos and enalaprilat—a study on enzyme kinetics. *Phytotherapy Research*.

[17] Rang H. P., Dale M. M., Ritter J. M., Moore P. K. (2007). *Rang & Dale's Pharmacology*.

[18] Ratnasooriya W. D., Fernando T. S. P. (2009). Anti-inflammatory activity of Sri Lankan black tea (*Camellia sinensis* L.) in rats. *Pharmacognosy Research*.

